# Group-1 Grass Pollen Allergens with Near-Identical Sequences Identified in Species of Subtropical Grasses Commonly Found in Southeast Asia

**DOI:** 10.3390/medicina55050193

**Published:** 2019-05-22

**Authors:** Sirirat Aud-in, Koravit Somkid, Wisuwat Songnuan

**Affiliations:** 1 M.Sc. Programme in Plant Sciences, Faculty of Graduate Studies, Mahidol University, Nakhon Pathom 73170, Thailand; sirirat.aud@student.mahidol.edu; 2 Department of Plant Science, Faculty of Science, Mahidol University, Bangkok 10400, Thailand; 3 Department of Pharmaceutical Botany, Faculty of Pharmacy, Bangkok 10400, Thailand; 4 Toxicology graduate programme, Faculty of Science, Mahidol University, Bangkok 10400, Thailand; koravit.sok@gmail.com; 5 Systems Biology of Diseases Research Unit, Faculty of Science, Mahidol University, Bangkok 10400, Thailand

**Keywords:** group-1 grass allergens, allergic rhinitis, pollen, subtropical grasses, beta-expansin

## Abstract

*Background and objectives:* Group-1 grass allergens or beta-expansins (EXPBs) are major allergens from pollen of all grass species. Previous studies showed that they are highly conserved (64–85%) in Pooideae species, which are found mostly in the temperate regions. However, the information about group-1 allergens from common grass species in subtropical areas is still lacking. This study aimed to assess the sequence diversity of group-1 grass pollen allergens in subtropical areas, especially in Southeast Asia. *Materials and Methods:* Group-1 allergens were cloned from pollen of eight grass species using a single set of primers. Sequences were analyzed and IgE and IgG_4_ binding regions were compared to the previously reported epitopes in homologous EXPBs. The phylogenetic analysis was used to assess the relationship between sequences of these species and previously characterized EXPBs. Moreover, three-dimensional structure of the EXPB was modeled based on homology to Zea m 1. *Results:* Sequences from eight grass species were nearly identical. It is conceivable that the primers used for cDNA amplification detected the same isoform in different species. In fact, the deduced amino acid sequences shared 97.79–100% identity with each other and 15/819 polymorphic nucleotide positions were identified. The predicted structure showed that the IgE and IgG_4_ epitopes and polymorphic residues were located in both domains 1 and 2. The dendrogram presents clustering of class A EXPBs into four groups corresponding to the grass subfamilies. *Conclusions*: This study identified the allergens with near-identical sequences from different grass species. This isoform could be the major cross-reacting allergenic protein from commonly found grass species.

## 1. Introduction

Group-1 grass allergens have been defined as major allergens of grass pollen on the basis of high prevalence and potency. The group-1 allergens caused IgE reactivity in more than 90% of grass pollen allergic patients [1]. These allergens have been identified in all grass species, unlike the group-5 allergens that are restricted to grasses of the Pooideae subfamily. In the tropical/subtropical areas where the Panicoideae and Chloridoideae grasses predominate, and temperate trees such as birch and beech are absent, group-1 grass allergens become the most relevant allergens for pollen allergy sufferers.

Group-1 allergens have been reported from several grass species. Phl p 1 from *Phleum pratense* (timothy grass) is among the most extensively studied allergens from the Pooideae grasses. Phl p 1 has high IgE reactivity among grass pollen allergic patients and it has been suggested that Phl p 1 together with Phl p 5 and profiling could be sufficient for a grass pollen allergy diagnosis in the temperate regions [2,3]. In subtropical areas, Cyn d 1, from *Cynodon dactylon* (Bermuda grass) is well-characterized, along with Pas n 1 from *Paspalum notatum* (bahia grass), Lol p 1 from *Lolium perenne* (ryegrass), Zea m 1 from *Zea mays* (maize), and Sor h 1 from *Sorghum halepense* (Johnson grass).

The expansin superfamily contains a large number of 31–35 kDa glycoproteins divided into the following four families based on sequence analysis: alpha-expansin (EXPA), beta-expansin (EXPB), expansin-like A (EXLA), and expansin-like B (EXLB) [4]. All group-1 allergens are classified as a subclass of EXPBs. Expansins are involved in extension and loosening of the extracellular matrix in the plant cell wall [5]. Their biological functions involve pollen tube growth through the female flower. The structure study of Zea m 1 showed that it is composed of two domains: The N-terminal domain 1 that resembles the catalytic domain of family 45 glycoside hydrolases (GH45) and domain 2 that consists of β-sheets with 36% sequence identity to group 2 and 3 grass pollen allergens [6].

Previous studies mostly focused on the characterization of group-1 grass allergens from temperate and subtropical grasses widely distributed in Europe or North America. The group-1 grass allergens are highly conserved, sharing 60–70% sequence identity [7]. Conservation of group-1 grass allergens within each grass subfamily can be even higher, as in the case of Phl p 1, the group-1 allergen of timothy grass that shared 85–95% identity at the amino acid level with EXPBs from other Pooideae species. This high sequence identity leads to high cross-reactivity among grass species [8]. However, information about other grass species is still limited. In subtropical and tropical regions where hundreds of grass species are present and the pollen season is not well-defined, identifying the primary source of grass pollen sensitization is a complicated task. Skin-prick testing with extracts from pollen of all common grass species without other supporting information is not an ideal approach. To help circumvent this problem, it was hypothesized that the sequence identity of grass group-1 allergens or EXPBs from common grass species could help in predicting the degree of cross-reactivity with known allergenic grass pollen species.

This study expands the understanding of sequence diversity of group-1 grass allergens from pollen of subtropical grasses frequently found in Southeast Asia. This is among the first studies to compare beta-expansin sequences obtained from multiple grass species using a single set of primers. We found that these primers yielded PCR products in eight out of ten selected species. Furthermore, all sequences from these eight species were nearly identical. The IgE and IgG_4_ binding epitopes and protein structure features were predicted. Clustering analysis was performed to determine the relationship between the sequences obtained in this study and previously reported group-1 allergens. This information is crucial in predicting the contribution of the isoform to the overall allergenicity of the pollen from each grass species, which may lead to the improvement of diagnosis and allergy immunotherapy for grass pollen allergy in subtropical areas, especially in Southeast Asia.

## 2. Materials and Methods

### 2.1. Plant Materials

Ten grass species were collected from several sites around Bangkok and metropolitan areas (Table 1). All grass species were identified based on the Key to Flora of North America (Wipff and Thompson, no date) and voucher specimens were preserved by plant taxonomy specialists at the Department of Plant Science, Faculty of Science, Mahidol University, Bangkok, Thailand.

Grass inflorescences were collected from natural sites. To avoid contamination, inflorescences were gathered from areas with few or no other species in close proximity and only a single grass species was processed during each period. Inflorescences were arranged in vessels and allowed to naturally release pollen for one day in a semi-closed area. Pollen grains along with other plant parts were gathered and passed through sieves to obtain pollen grains with high purity. The purity was accessed under a light microscope. Only the pollen samples with a purity of >95% were used. The percentage of pollen purity was calculated as follows: (no. grass pollen grains/[no. grass pollen grains + no. other contaminants]) × 100 = x%. The pollen grains were stored at −80 °C until used.

### 2.2. RNA Extraction and cDNA Synthesis

For RNA extraction, 100 mg of pollen grains kept at −80 °C were ground to fine powder with liquid nitrogen. Total RNA was isolated using 1 mL TRIzol™ reagent (Invitrogen, Carlsbad, CA, USA) according to the manufacturer’s protocol. The RNA quality was assessed using agarose gel electrophoresis, and the RNA concentration was measured using a NanoDrop 2000 spectrophotometer (Thermo Scientific, Waltham, MA, USA). Reverse transcription was performed using an iScript™ Select cDNA synthesis kit (Bio-Rad, Hercules, CA, USA).

### 2.3. RACE PCR Cloning

The rapid amplification of cDNA end (RACE) PCR was conducted using beta-expansin specific primers and a cDNA template from *Cynodon dactylon*, *Zoysia matrella*, *Sorghum halepense*, and *Bothriochloa pertusa*. The 5′ and 3′ RACE cDNA from 1 ug of total RNA was separately synthesized using a SMARTScribe reverse transcriptase (Clontech Laboratories, Mountain View, CA, USA). Universal primer A mix and gene-specific primers were used for amplification: KS-CD1/2 (F: 5′-gattacgccaagcttgccgggccccaacatcactgcaacctac-3′, R: 5′-gattacgccaagcttttggacttgtagacggtgtcgggcttcc-3′) for *C. dactylon* and KS-ZM1/2 (F: 5′-gattacgccaagcttggggtgcggcaacgagcccatc-3′, R: 5′-gattacgccaagcttaagtttggcgccgctctcgctggt-3′) for *Z. matrella*, *S. halepense*, and *B. pertusa*. The amplification was conducted using touchdown PCR as described in the kit manual.

### 2.4. Cloning and Sequencing

The beta-expansin (EXPB) cDNAs were amplified by PCR using Vivantis^®^ Taq DNA polymerase (Vivantis, Subang Jaya, Malaysia). The specific primers KS-4.2/p3 (F: 5′-cacatcacattacacagcaggagaaag-3′, R: 5′-ctctaccgacttgtgtgcg-3′) were designed from highly conserved regions of the RACE PCR products. These primers were used for PCR amplification. The amplicons were analyzed by agarose gel electrophoresis and purified by a QIAquick^®^ gel extraction kit (Qiagen, Redwood City, CA, USA). For TA cloning, dATPs were added into the purified PCR products for elongation of the A-tail. The resulting fragments were ligated into pGEM^®^-T easy vector (Promega, Medison, WI, USA). Transformation into competent *E. coli* DH5α cells was performed using the heat-shock method. For each species, twenty positive clones were picked and colony PCR was performed to select clones with insertions. Selected clones (3–4 clones/species) were cultured overnight and plasmids were extracted from precipitated cells using QIAprep^®^ spin miniprep kit (Qiagen, Germantown, MD, USA). Sanger sequencing was conducted based on the plasmid template by a commercial laboratory (Macrogen, Korea) using M13 primers.

### 2.5. Sequence Analysis

The nucleotide sequences from forward and reverse sequencing were checked for quality and accuracy based on the electropherograms using BioEdit software [17]. Only sequences of good quality (read length > 1000 bases) were included in the analysis. All sequences were trimmed to begin at the start codon and end at the stop codon prior to the sequence analysis. No insertion/deletion was observed in the consensus sequences. For each species, the clone with the highest percent identity to the consensus sequence was chosen for the intra-species sequence comparison. The nucleotide sequences were subjected to BLASTn homology search using a nucleotide collection (nr/nt) database with Megablast (optimized for highly similar sequences). Deduced amino acid sequences were obtained using the ExPaSy translate tool using standard parameters. Multiple sequence alignments of nucleotide and amino acid sequences were constructed and analyzed using BioEdit. The percent identity was calculated using the Clustal Omega program [18]. The program parameters were set as default: ClustalW with character counts (clustal_num), dealign input sequences (no), mBed-like clustering guide-tree (yes), mBed-like clustering iteration (yes), number of combined iterations (default 0), maximum guide tree iterations (default), maximum HMM iterations (default), and order (align). The percent identity matrix was created using Clustal 2.1 in Clustal Omega program.

### 2.6. Protein Structure Prediction

The IgE and IgG4 binding epitopes corresponding to Cyn d 1 epitopes were based on previous studies [19,20,21,22]. The conserved catalytic sites of family 45 glycoside hydrolases (GH45) enzymes were also predicted based on sequence homology to Zea m 1 [6]. The three-dimensional structure of EXPB was constructed using a template from an automated protein homology-modeling server (SWISS-MODEL, Basel, Switzerland) [23]. The Zea m 1 structure used as a template was extracted from PDB protein databank. The image was generated using the PyMOL molecular graphics system (DeLano Scientific, Palo Alto, CA, USA).

### 2.7. Phylogenetic Analysis

EXPB sequences of eight grass species in this study were compared to other species from the allergen database reported by the WHO/IUIS. A phylogenetic tree was constructed by MEGA7 [24] using the neighbor-joining method [25]. The phylogeny was tested using the bootstrap method with 100 replicates. The evolutionary distances were computed using the Poisson correction method [26].

## 3. Results

We assessed the diversity of group-1 grass allergen or beta-expansin (EXPB) sequences from grass species commonly found in subtropical areas such as Thailand. The ten selected species are shown in Table 1. Because the genomes of these grasses were not available, the consensus sequence from RACE-PCR was used for designing the beta-expansin specific primers. One primer pair was used to successfully clone EXPB from eight out of ten selected species. Several clones were obtained from each species (Supplementary Materials Table S1) and only the sequences of good quality were used for further analysis. Not all of the sequences from the same species were identical (percent identity ranged from 95.24–100%). The sequences with the highest percent identity to the consensus sequence was used for inter-species sequence analysis. The amplicons from all eight species had an identical length of 819 base pairs. The full-length encoding regions were translated to 272 amino acids with an approximated molecular weight of 30 kDa, similar to other previously reported EXPBs.

Multiple-sequence alignment showed that the EXPBs obtained from these eight species were remarkably similar, even though no two sequences were identical. The percent identity of nucleotide and amino acid sequences is presented in Table 2. At the nucleotide level, the highest percent identity was 99.88% between Pol i EXPB and Bot p EXPB. Only 1/819 nucleotide was different between these two species. The lowest percent identity (98.78%) was found between Bot p EXPB and Eri p EXPB with ten variations. In total, 15 polymorphic positions were identified. The deduced amino acid sequences of EXPB from the eight species shared 97.79–100% identity with each other. Notably, the deduced amino acid sequence of Zoy m EXPB had a 100% match with that of Pol i EXPB.

Identity analysis of nucleotide and deduced amino acid sequences was performed by Clustal Omega [18]. The columns indicate nucleotide sequence identity, and the rows indicate deduced amino acid sequence identity. The numbers 1–8 represent eight common grasses.

**Figure 1:**
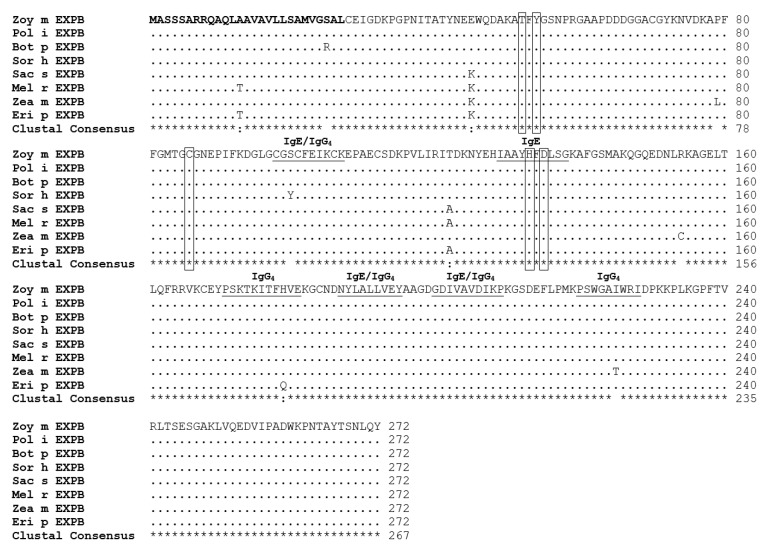
Multiple sequence alignment of deduced amino acid sequences of EXPB from eight common grasses. Sequence alignment was performed using the BioEdit program [17]. Dots represent amino acids that were identical to Zoy m EXPB. Bold residues indicate signal peptide. Rectangular frames indicate catalytic sites identified in GH45 enzymes corresponding residues of EXPB1 (Zea m 1) and EXPB in this study. The IgE and IgG_4_ binding epitopes were marked by underlines [6,19,20,21]. The nucleotide sequences obtained in this study were submitted to GenBank with accession numbers as follows: Zoy m EXPB: MK393185, Pol I EXPB: MK393186, Bot p EXPB: MK393187, Sor h EXPB: MK393188, Sac s EXPB: MK393189, Zea m EXPB: MK393190, Mel r EXPB: MK393191, Eri p EXPB: MK393192.

Alignment of deduced amino acid sequences (Figure 1) showed six variations between Zea m EXPB and Eri p EXPB sequences, leading to the lowest percent identity at 97.79%. In all eight sequences, only nine polymorphic residues were identified (at positions 13, 25, 45, 79, 100, 122, 154, 179, and 225). All sequences contained an identical predicted signal peptide (26 aa) at the N-terminus. The residues that corresponded to the catalytic sites of the family 45 glycoside hydrolases (GH45) enzymes at T52, Y54, C86, H133, and D135 were completely conserved in all sequences. On the basis of the previously characterized human IgE and IgG_4_ binding epitopes of Cyn d 1 [19,20,21,22], the corresponding epitopes were found in all species. Three changes were observed within these predicted IgE and IgG_4_ binding epitopes: S100Y, H179Q, and I225T. The experimental exchangeability for these residues were 0.173, 0.396, and 0.198, respectively [27], suggesting that the H179Q may have had less effect than S100Y and I225T.

**Figure 2:**
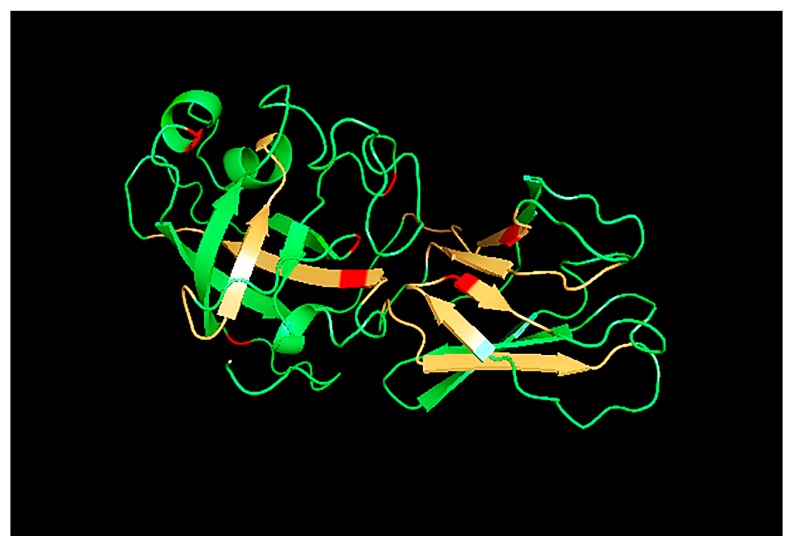
Predicted structure of Zoy m EXPB. The human IgE and IgG_4_ binding sites of Cyn d 1 were shown in the three-dimensional structure of EXPB from *Zoysia matrella* (orange). The red residues indicate polymorphisms in eight grass species sequences. The known structure template was crystal structure of EXPB1 (Zea m 1) [6] from an automated protein homology-modeling server (SWISS-MODEL) [23]. The image was generated using the PyMOL molecular graphics system (DeLano Scientific).

To gain better insights into the observed polymorphisms, the Zoy m EXPB sequence was used for homology modeling. The crystal structure of beta-expansin protein Zea m 1, with 62.61% identity to the Zoy m EXPB sequence, was selected from PDB as a template model [6]. Figure 2 shows the three-dimensional model structure of Zoy m EXPB. Similar to Zea m 1 structure, Zoy m EXPB contains two domains. Domain 1 consists of a six-stranded β-barrel, short loops, and two α-helices. This domain contains the predicted catalytic site of GH45 enzymes. Domain 2 is composed of eight β-sheets, connected to Domain 1 by a short linker. Five of seven polymorphic residues are found in Domain 1, one in β-barrel, one in α-helix, and three in loop regions. The other two polymorphic residues in Domain 2 are located in the β-sheet region. The predicted IgE/IgG_4_ binding epitopes are located in both domains and are mostly exposed on the protein surface.

**Figure 3:**
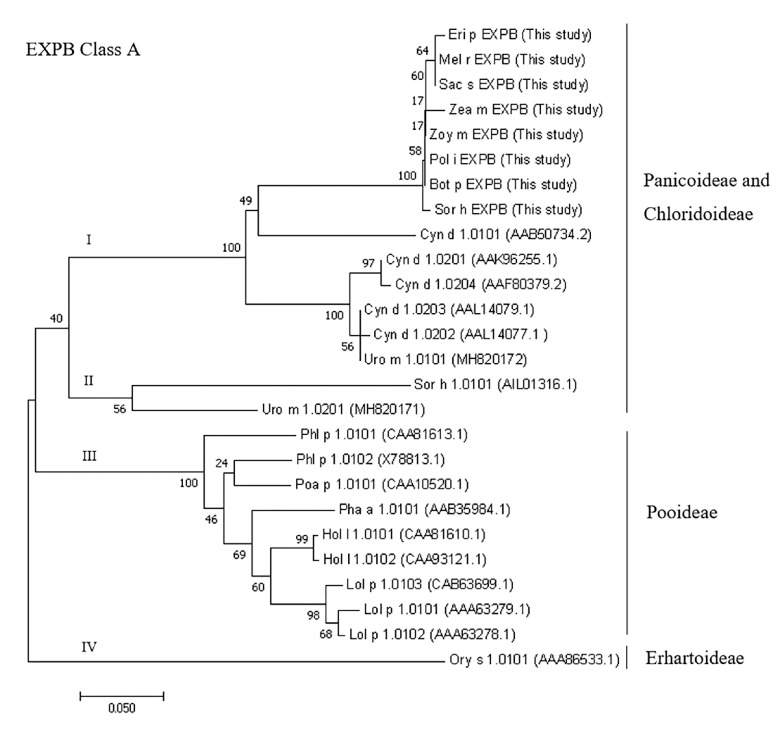
The phylogenetic tree of protein sequences from this study and other grass group 1 pollen allergens. The evolutionary history was constructed in MEGA7 [24] using the neighbor-joining method [25]. The stability of the tree was supported by a bootstrap test with 1000 replicates. The evolutionary distances were computed using the Poisson correction method [26]. Allergen sequences were obtained from the Allergen Nomenclature database and the homologous protein sequences from Blastp analysis. The GenBank accession numbers are indicated after the allergen names.

To assess the relationship of EXPBs obtained from the eight species in this study and previously reported species, a phylogenetic tree was constructed using the neighbor-joining method. Eighteen previously characterized group-1 grass allergens with high (>57%) identity to sequences obtained in this study were retrieved from the GenBank database. The resulting dendrogram (Figure 3) shows that EXPBs could be divided into four subgroups largely corresponding to the grass subfamilies: (I) and (II) Panicoideae and Chlorodoideae subfamilies, (III) Pooideae subfamily, and (IV) Erhartoideae subfamily. Eight allergens from this study were clustered together in subgroup I, with Cyn d 1 (all isoforms) and Uro m 1.0101 as the most closely related sequences. Subgroup II was composed of Sor h 1 and Uro m 1. Beta-expansins from Pooideae grasses were clustered into subgroup III and Ory s 1 was separated into subgroup IV.

## 4. Discussion

On the basis of the previously reported EXPBs, the expected percent identity between sequences from different grass species is in the range of 64–85% [28]. Surprisingly, this study identified a near-identical (97.79–100%) isoform of EXPBs from eight out of ten selected grass species. Furthermore, this is the first study to report two identical EXPBs from pollen of different grass species.

One possible reason that this near-identical isoform was not characterized previously is that most studies focused on characterization of isoallergens from a single grass species such as Sor h 1 from Johnson grass (2 isoforms) [29], Lol p 1 from rye grass (3 isoforms) [30,31], Hol l 1 from velvet grass (2 isoforms) [32,33], Phl p 1 from timothy grass (2 isoforms) [2,34], and Cyn d 1 from Bermuda grass (5 isoforms) [19,21,35]. The highest sequence identity between isoforms isolated from the same species was found with Cyn d 1 (86.4–99.6%) [18,21]. Because these species were investigated independently, the isoforms were obtained using different conditions. In particular, the PCR primers were designed from different sequences. Hence, the resulting products might not be derived from genes with the highest percent identity. In this study, a common primer pair was used to obtain the PCR products from several species, allowing amplification of the cDNA from the most similar orthologous genes.

On the basis of the clustering analysis, Cyn d 1.0101 (accession no. AAB50734.2), a major allergen from Bermuda grass, is the previously reported allergen most closely related (81.15–81.97%) to the near-identical isoform found in this study. This Cyn d 1 isoform has been shown to have a high frequency of IgE reactivity in grass pollen allergic patients and cross-reacted with Phl p 1 [36,37].

It is likely that the near-identical isoform will have similar IgE reactivity to the Cyn d 1.0101, although a few polymorphisms found within the predicted IgE and IgG_4_ epitopes could affect its allergenic potential [21,22].

The near-identical EXPB isoform in this study was cloned from grasses across two grass subfamilies: Chloridoideae (Zoy m EXPB) and Panicoideae (seven other EXPBs), suggesting that this isoform could be considerably prevalent in the grass family rather than limited to closely related taxa. Due to its high percent identity, this isoform could be the major cross-reacting allergenic protein between pollen of commonly found grass species. Nonetheless, the contribution of this isoform to the total allergenic potency of the pollen should be further investigated, since it also depends on the expression level and accessibility of this protein in the context of other EXPBs and other major and minor allergens.

This study provides supporting evidence that allergen isoforms from different species can have sequences that are more similar than (or identical to) isoforms within the same species. This situation warrants further discussion of the current IUIS Allergen Nomenclature Sub-Committee guideline suggesting that isoallergens are allergens from a single species with >67% sequence identities, and variants of an isoallergen are defined as proteins with >90% sequence identity [38]. Perhaps an additional term such as “orthoallergen” or “homoallergen” should be used to designate identical or near-identical allergens identified from different source species. As more genomic and proteomic data become available, the identification of these identical allergens would be increasingly simple and widespread in the near future.

## 5. Conclusions

This study expands the understanding of sequence diversity of group-1 grass pollen allergens from subtropical areas. A group-1 allergen isoform was identified from different grass species with high sequence identity. This isoform could be the major cross-reacting allergenic protein between these species. Further investigation of IgE binding of this isoform, especially in comparison with the existing isoforms of beta-expansin, should provide critical information for diagnosis and allergen-specific immunotherapy for subtropical grass pollen allergy.

## Figures and Tables

**Table 1:** Common grass species in Thailand selected for this study.

Scientific Name	Common Name	Reported Allergenicity (Patient Group)	Near Identical EXPB (This Study)	GPS Coordinates
*Zoysia matrella* (L.) Merr.	Manila grass	No report	Zoy m EXPB	14.079971, 99.702437
*Polytrias indica* (Houtt.) Veldkamp	Batiki bluegrass	No report	Pol i EXPB	13.850821, 100.527323
*Bothriochloa pertusa* (L.) A.Camus	Hurricane grass	No report	Bot p EXPB	14.113808, 99.150451
*Sorghum halepense* (L.) Pers.	Johnson grass	77% (48 GP- allergic), 21% (100 AR with + SPT to common inhalant allergens), 10% (419 AR) [9,10,11]	Sor h EXPB	14.047468, 99.780618
*Saccharum spontaneum* L.	Canne sauvage	No report	Sac s EXPB	13.921569, 100.490671
*Zea mays* L.	Maize, corn	6.25% (48 patients suspected to have nasobronchial allergy) [12]	Zea m EXPB	14.071024, 99.729950
*Melinis repens* (Willd.) Zizka	Natal grass	No report	Mel r EXPB	14.129449, 99.161192
*Eriochloa procera* (Retz.) C.E.Hubb.	Cup grass	No report	Eri p EXPB	13.850821, 100.527323
*Urochloa mutica* (Forssk.) T.Q.Nguyen	Para grass	53.2% (2,383 AR with + SPT to pollen) [13]	-	13.740637, 99.920501
*Cynodon dactylon* (L.) Pers.	Bermuda grass	84% (48 GP-allergic and AR), 2.1% (419 AR), 12.5% (48 GP- allergic and AR), 54.9% (2,383 AR), 61% (54 Perennial AR), 20.5% (200 As), 52% (133 GP-allergic with + IDST) [9,11,12,13,14,15,16]	-	14.075435, 99.698798

GP: grass pollen, AR: allergic rhinitis, As: asthma, SPT: skin prick test, IDST: intradermal skin test, +: positive result.

**Table 2:** Percent identity of nucleotide and deduced amino acid sequences of EXPB from eight common grasses.

	Amino Acid									
	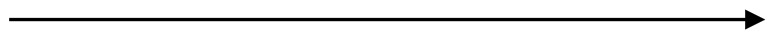									
		1	2	3	4	5	6	7	8	
Nucleotide 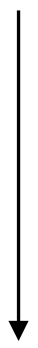	1	**100.00**	100.00	99.63	99.63	99.26	98.90	98.53	98.53	1. Zoy m EXPB
	2	99.76	**100.00**	99.63	99.63	99.26	98.90	98.53	98.53	2. Pol i EXPB
	3	99.39	99.63	**100.00**	99.26	98.90	98.53	98.16	98.16	3. Bot p EXPB
	4	99.63	99.88	99.51	**100.00**	98.90	98.53	98.16	98.16	4. Sor h EXPB
	5	99.63	99.39	99.02	99.27	**100.00**	99.63	98.53	99.26	5. Sac s EXPB
	6	99.63	99.39	99.02	99.27	99.76	**100.00**	98.16	99.63	6. Mel r EXPB
	7	99.51	99.27	98.90	99.15	99.39	99.39	**100.00**	97.79	7. Zea m EXPB
	8	99.39	99.15	98.78	99.02	99.51	99.76	99.15	**100.00**	8. Eri p EXPB

## Supplementary Materials

The following are available online at https://www.mdpi.com/1010-660X/55/5/193/s1, Table S1: Nucleotide sequences from cDNA clones of eight grass species.
