# Supplementary material for: Group-1 Grass Pollen Allergens with Near-Identical Sequences Identified in Species of Subtropical Grasses Commonly Found in Southeast Asia
Source: Medicina (Kaunas). 2019 May 22;55(5):193. doi: 10.3390/medicina55050193 (PMC6571983; doi:10.3390/medicina55050193)
Supplement: Supplementary file 1 [file medicina-55-00193-s001.pdf]

Supplemental Table S1 Nucleotide sequences from cDNA clones of eight grass species.

Grass species	No. of clones	Seq. identity among clones*	Homologous Cyn d 1 sequence[#]
Zo. matrella	4	99.76-100.0%	Cyn d 1.0203; AF177380.1 (85%)
P. indica	3	99.88-100.00%	Cyn d 1.0203; AF177380.1 (85%)
B. pertusa	4	99.33-99.51%	Cyn d 1.0203; AF177380.1 (85%)
S. halepense	4	99.20-99-30%	Cyn d 1.0203; AF177380.1 (86%)
S. spontaneum	2	100%	Cyn d 1.0203; AF177380.1 (85%)
Ze. mays	4	95.24-99.02%	Cyn d 1.0203; AF177380.1 (83%)
E. procera	4	97.43-100.00%	Cyn d 1.0203; AF177380.1 (85%)
M. repens	3	99.39-99.51%	Cyn d 1.0202; AF177378.1 (85%)

*, Sequence identity among clones was analyzed using Clustal Omega. [#]; Homologous sequences were analyzed using nucleotide Blastn analysis with more than 80% sequence identity. Percentages of sequence identities are presented in parentheses.
